# Effect of Copper on the Function of Isolated Porcine Kidneys Stored Using Simple Hypothermia

**DOI:** 10.3390/ijms232113031

**Published:** 2022-10-27

**Authors:** Aneta Ostróżka-Cieślik, Barbara Dolińska, Florian Ryszka

**Affiliations:** 1 Department of Pharmaceutical Technology, Faculty of Pharmaceutical Sciences in Sosnowiec, Medical University of Silesia, Kasztanowa 3, 41-200 Sosnowiec, Poland; 2 “Biochefa” Pharmaceutical Research and Production Plant, 3, 41-200 Sosnowiec, Poland

**Keywords:** copper, prolactin, kidney, preservation, perfusion

## Abstract

Renal ischemia in the peri-transplant period causes a number of changes that adversely affect the initiation of normal vital functions in grafts after transplantation. To minimise the extent of ischemic damage, organs are stored in preservation fluid. The components of the fluid are supposed to ensure stabilisation of the cell cytoskeleton, protect against oxygen free radicals, reduce cell swelling, and ensure endothelial cell integrity. The aim of this study was to analyse the protective effect of Cu^2+^, as a component of Biolasol preservative fluid, in the prevention of nephron damage occurring during the graft storage period. Analyses of the effectiveness of copper in the presence of prolactin added to Biolasol fluid were also carried out. Forty isolated pig slaughter kidneys were used in the study, avoiding the use of laboratory animals. The kidneys were stored using simple hypothermia. After 2 h and 48 h of graft storage, selected biochemical indicators of renal function were determined in the collected perfusates. The addition of Cu^2+^ at a dose of 1 µg/L to the composition of Biolasol fluid was found to affect the generation of ischemic damage in the isolated pig kidney. The intensity of the occurrence of these processes is exacerbated by the presence of prolactin at a dose of 0.1 µg/L.

## 1. Introduction

Renal ischemia in the peri-transplant period causes a number of changes that adversely affect the initiation of normal vital functions in grafts after transplantation. The mechanism of ischemic damage has been described in our previous articles [1,2]. The key point is that ischemia causes a rapid depletion of intracellular reserves of high-energy compounds (ATP). A lack of oxygen supply triggers anaerobic glycolysis, an increase in lactic acid concentration, and metabolic acidosis. Oxygen free radicals (ROS) are generated. The consequence of these processes is cell death. To minimise the extent of ischemic damage, organs are stored in preservation fluid. The fluid components are primarily designed to stabilise the cell cytoskeleton, protect against oxygen free radicals, reduce cell swelling, and ensure the integrity of endothelial cells. Our team is conducting advanced research in the field of optimising the composition of the preservative fluid. One of our research objectives is to gain knowledge of the antioxidant activity of selected bioelements added to Biolasol fluid. We have shown that selenium (Se^4+^) adversely affects renal function during ischemia-reperfusion. We obtained the best protection using Biolasol modified with selenium (IV) at a dose of 1 µg/L, alongside prolactin (PRL: 0.1 µg/L) [1]. Zinc added to Biolasol fluid showed little efficacy in protecting nephrons. In contrast, Zn^2+^ added to Biolasol + PRL fluid (0.1 µg/L) acted as a prolactin inhibitor [2]. In addition, manganese (Mn^2+^: 1 µg/L) did not show antioxidant activity as a component of Biolasol fluid [3]. We also investigated copper as another element with antioxidant potential in renal protection.

Copper is a micronutrient that is essential for the body to function properly. It is estimated that the highest amount of copper in the adult body is deposited in the liver (3.47 µg/g), brain (3.32 µg/g), heart (3.26 µg/g), kidney (2.15 µg/g), and bile (3.6 µg/mL) [4,5]. Copper exhibits oxidoreductive properties. It is involved in the regulation of the intracellular redox potential and is a component of proteins with enzymatic activity (e.g., Cu, Zn-superoxide dismutase, ceruloplasmin, cytochrome c oxidase (COX), tyrosinase, dopamine beta-monooxygenase) [6,7]. Copper deficiency results in reduced antioxidant defence. In turn, an increase in cellular copper concentration increases lipid peroxidation and depletes glutathione (GSH) reserves [8]. Copper transport across biological membranes is controlled by a group of proteins belonging to the copper transporter (CTR) and divalent metal-ion transporter-1 (DMT1) families. The main membrane protein involved in the uptake of Cu^+^ cations by the cell is CTR1 (copper transporter 1). The expression of this membrane protein has been observed in all types of tissues and organs. Another relevant protein is CTR2 (copper transporter 2), which is located in vesicular structures within the cytoplasm. In contrast, Cu^+^ and Cu^2+^ ions are capable of interacting directly with DMT1 [9,10]. ATPases (ATP7A and ATP7B) regulate the concentration of copper ions in the cell and mediate their incorporation into enzyme proteins [6]. The bioavailability of the element is regulated by Cu transport proteins including the cytosolic Cu chaperone antioxidant-1 (Atox1) [11].

Studies indicate that ischemic disease induces a decrease in copper ion concentration in ischemic myocardial tissue. At the same time, serum copper concentrations increase due to the upregulation of copper metabolism MURR domain 1 (COMMD1) in the heart upon ischemic insult [12]. Tural et al. [13] found a protective effect of copper (CuSO_4_, 0.1 mg Cu^2+^/kg/day) in the presence of betanin (100 mg/kg) in the heart and lung during ischemia-reperfusion injury in a rat model. The authors observed a decrease in malondialdehyde (MDA, one of the end products of lipid peroxidation) and myeloperoxidase (MPO, involved in inflammatory processes). Xiao’s team [14] confirmed that Cu supplementation (CuSO_4_, 0.2 mg Cu^2+^/kg with human serum albumin) protects endothelial cells from apoptosis in the ischemic myocardium in a mouse model. Cu-albumin administration up to 28 days after myocardial infarction resulted in the protection of myocardial contractile function. Copper homeostasis in the body has been observed to be impaired in patients with chronic kidney disease (CKD) and in haemodialysis patients [15,16,17]. Singh et al. [18] observed a decrease in copper-zinc superoxide dismutase activity in rat kidneys during ischemia-reperfusion injury. In contrast, other authors have suggested that Cu as a component of superoxide dismutase and ceruloplasmin may counteract oxidative injury [19,20,21].

Recently published research papers [13,14,15,16,17,18,19,20,21] suggest the potential of copper in protecting the kidney during ischemia. The aim of our study was to analyse the protective effect of Cu^2+^, as a component of Biolasol preservative fluid, in the prevention of nephron damage occurring during graft storage using simple hypothermia. We also conducted analyses of the efficacy of copper in the presence of prolactin (PRL) added to Biolasol fluid. The results of our previous studies suggest an effective action of PRL in protecting the kidney during ischemia [22,23].

## 2. Results

**Figure 1:**
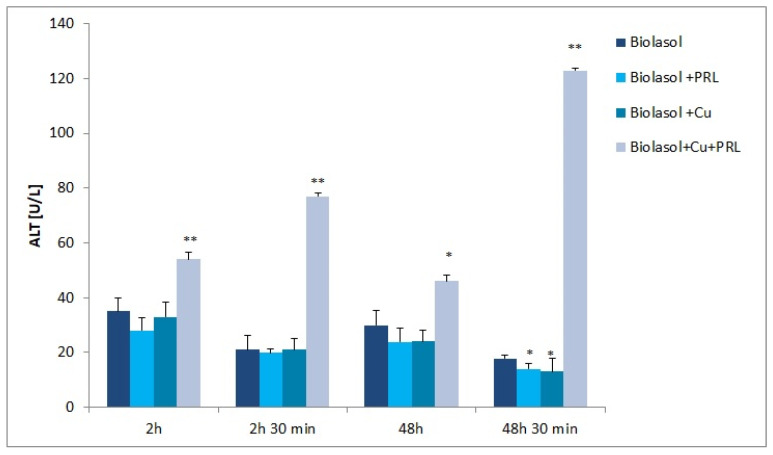
ALT activity in collected perfusate samples. The values are expressed as mean ± SEM. Data were analysed by one-way ANOVA and Bonferroni post hoc tests; n = 10; * *p* < 0.05; ** *p* < 0.01 compared to the control group (Biolasol).

**Figure 2:**
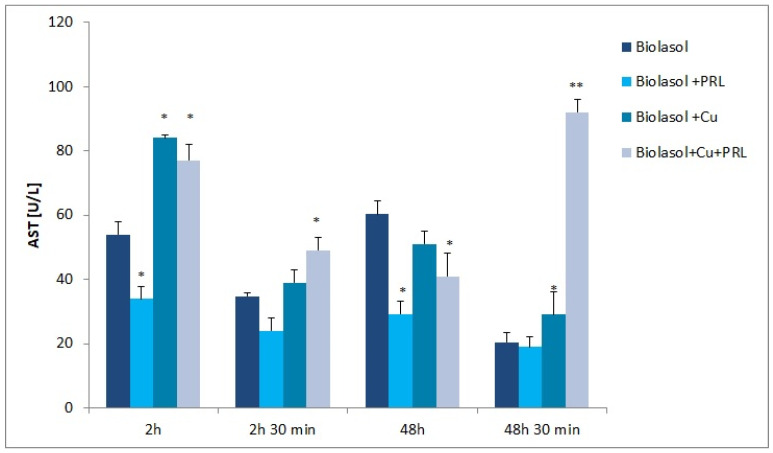
AST activity in collected perfusate samples. The values are expressed as mean ± SEM. Data were analysed by one-way ANOVA and Bonferroni post hoc tests; n = 10; * *p* < 0.05; ** *p* < 0.01 compared to the control group (Biolasol).

**Figure 3:**
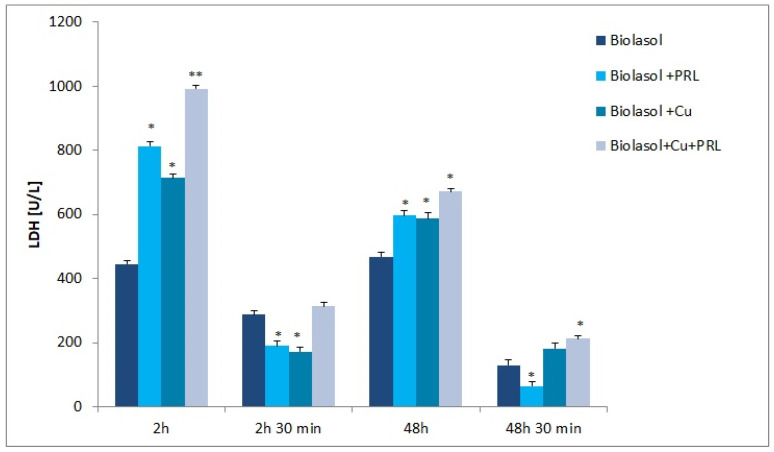
LDH activity in collected perfusate samples. The values are expressed as mean ± SEM. Data were analysed by one-way ANOVA and Bonferroni post hoc tests; n = 10; * *p* < 0.05; ** *p* < 0.01 compared to the control group (Biolasol).

**Figure 4:**
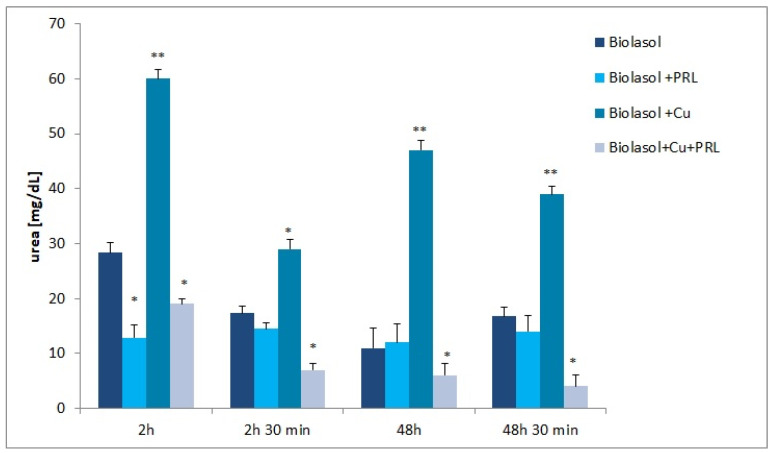
Urea concentration in the perfusate samples collected. The values are expressed as mean ± SEM. Data were analysed by one-way ANOVA and Bonferroni post hoc tests; n = 10; * *p* < 0.05; ** *p* < 0.01 compared to the control group (Biolasol).

**Figure 5:**
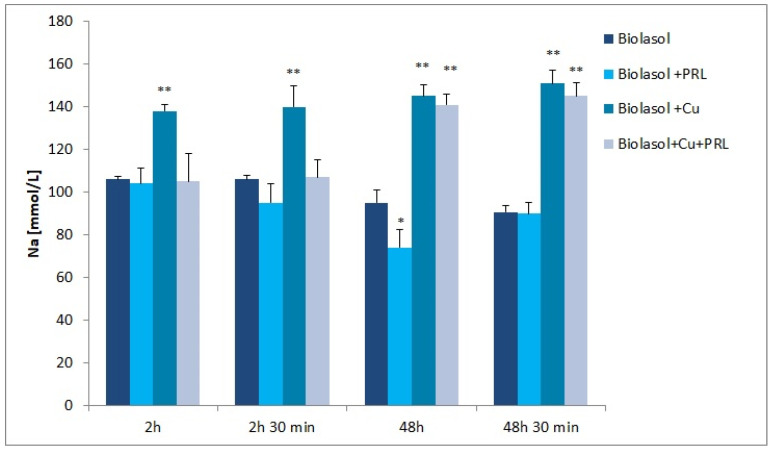
Sodium concentration in perfusate samples taken. The values are expressed as mean ± SEM. Data were analysed by one-way ANOVA and Bonferroni post hoc tests; n = 10; * *p* < 0.05; ** *p* < 0.01 compared to the control group (Biolasol).

**Figure 6:**
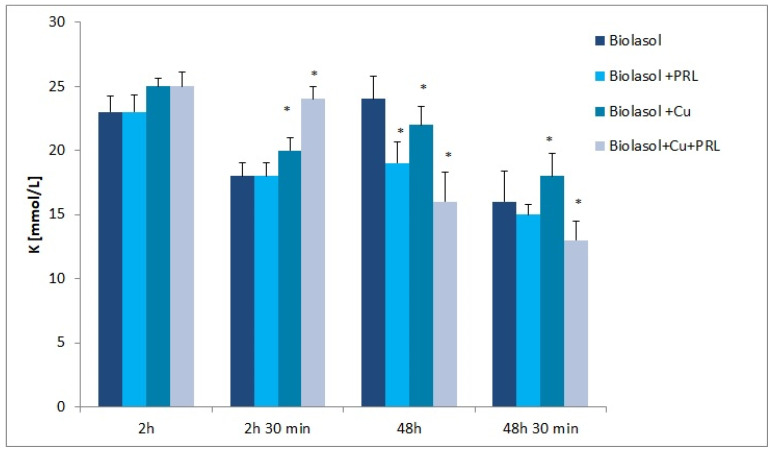
Potassium concentration in the perfusate samples collected. The values are expressed as mean ± SEM. Data were analysed by one-way ANOVA and Bonferroni post hoc tests; n = 10; * *p* < 0.05 compared to the control group (Biolasol).

The dynamics of changes in indicators of renal damage during perfusion and preservation are shown in Figure 1, Figure 2, Figure 3, Figure 4, Figure 5 and Figure 6. The increases in transaminases (AST and ALT) and lactate dehydrogenase activity are proportional to the degree and extent of graft damage following ischemia. ALT is an indicator enzyme of cytoplasmic origin. AST is a marker located mainly in the cytoplasm (20% of total activity) and mitochondria of cells (80% of total activity), and in renal epithelial cells. In contrast, lactate dehydrogenase (LDH) is an enzyme belonging to the oxidoreductase class and has been found in the cytoplasm of all cells. An increase in LDH activity is observed in disease states with tissue necrosis and in acute organ damage, including of the kidney [24,25].

The data illustrated in Figure 1 show that ALT activity varied during graft storage. Kidneys stored in Biolasol + Cu^2+^ + PRL fluid (B3 group) released the most ALT. The peak of activity appeared after 48 h 30 min (123 ± 1 U/L; *p* < 0.01) and was 591% higher versus the Biolasol group. ALT activity levels for this group were also higher at other time points by, respectively: 2 h—B3 group versus A group increased by 54% (*p* < 0.01); 2 h 30 min—B3 group versus A group increased by 267% (*p* < 0.01); 48 h—B3 group versus A group increased by 53% (*p* < 0.05). The activities of this marker in the perfusates of the B1 and B2 groups versus the control group, after 2 h, 2 h 30 min, and 48 h, respectively, were lower, although not statistically significant. ALT activity levels after 48 h 30 min were lower in the perfusates of the Biolasol + Cu^2+^ versus Biolasol group by 27% (*p* < 0.05).

A similar relationship was observed when analysing AST activity in the collected perfusates (Figure 2). The maximum aspartate aminotransferase activity was observed in the Biolasol + Cu^2+^ + PRL group after a time of 48 h 30 min (activity increase of 349% versus Biolasol, *p* < 0.01). Biolasol fluid modified with copper resulted in an increase in AST activity after time: 2 h renal storage (56% versus Biolasol, *p* < 0.05), 2 h 30 min perfusion (12% versus Biolasol, *p* = ns) and 48 h 30 min (41% versus Biolasol, *p* < 0.01).

LDH activity was also significantly increased in the Biolasol + Cu^2+^ + PRL group (Figure 3). The activity level of this indicator increased in perfusates taken at certain time points, respectively: 2 h—B3 group versus A group increased by 123% (*p* < 0.01); 48 h—B3 group versus A group increased by 43% (*p* < 0.05); 48 h 30 min—B3 group versus A group increased by 63% (*p* < 0.05). At 2 h 30 min, the LDH activity in the B3 group was similar to the level of the marker in the control group (*p* = ns). The addition of copper to Biolasol fluid significantly affected lactate dehydrogenase efflux from cells: 2 h—B2 group versus A group increased by 49% (*p* < 0.05); 48 h—B2 group versus A group increased by 25% (*p* < 0.05); 48 h 30 min—B3 group versus A group increased by 40% (*p* = ns). The presence of copper in the system, Cu^2+^ + prolactin or Cu^2+^ in the composition of Biolasol, adversely affects the integrity of the mitochondrial and cytoplasmic membranes of cells.

Figure 4 shows the results of urea determination. The lowest concentration of this marker was determined in the Biolasol + Cu^2+^ + PRL perfusates. Its concentration was correspondingly lower in the following groups: 2 h—B3 group versus A group decreased by 33% (*p* < 0.05); 2 h 30 min—B3 group versus A group decreased by 60% (*p* < 0.05); 48 h—B3 group versus A group decreased by 45% (*p* < 0.05); 48 h 30 min—B3 group versus A group decreased by 76% (*p* < 0.05). The addition of copper to the Biolasol formulation had a statistically significant effect on the increase in urea concentration. A markedly increased concentration of this indicator was found as early as 2 h into renal preservation (B3 group versus A group increased by 112%, *p* < 0.05), which was maintained throughout the period of perfusion and graft preservation: 2 h 30 min—B3 group versus A group increased by 68% (*p* < 0.05); 48 h—B3 group versus A group increased by 327% (*p* < 0.05); 48 h 30 min—B3 group versus A group increased by 132% (*p* < 0.05). The presence of copper in the system, Cu^2+^ + prolactin or Cu^2+^ in the Biolasol formulation, has a negative effect on graft filtration.

Sodium ion concentrations (Figure 5) increased gradually in the Biolasol + Cu^2+^ group (2 h—138 ± 6 mmol/L; 2 h 30 min—140 ± 8 mmol/L; 48 h—145 ± 5 mmol/L; 48 h 30 min—151 ± 5 mmol/L) and Biolasol + Cu^2+^ + PRL group (2 h—105 ± 3 mmol/L; 2 h 30 min—107 ± 5 mmol/L; 48 h—141 ± 6 mmol/L; 48 h 30 min—145 ± 6 mmol/L). In contrast, potassium ion concentrations gradually decreased (Figure 6). [K^+^] Biolasol + Cu^2+^: 2 h—25 ± 2 mmol/L; 2 h 30 min—22 ± 2 mmol/L; 48 h—20 ± 1 mmol/L; 48 h 30 min—18 ± 2 mmol/L. [K^+^] Biolasol + Cu^2+^ + PRL: 2 h-25 ± 3 mmol/L; 2 h 30 min—24 ± 1 mmol/L; 48 h—16 ± 2 mmol/L; 48 h 30 min—13 ± 2 mmol/L).

**Figure 7:**
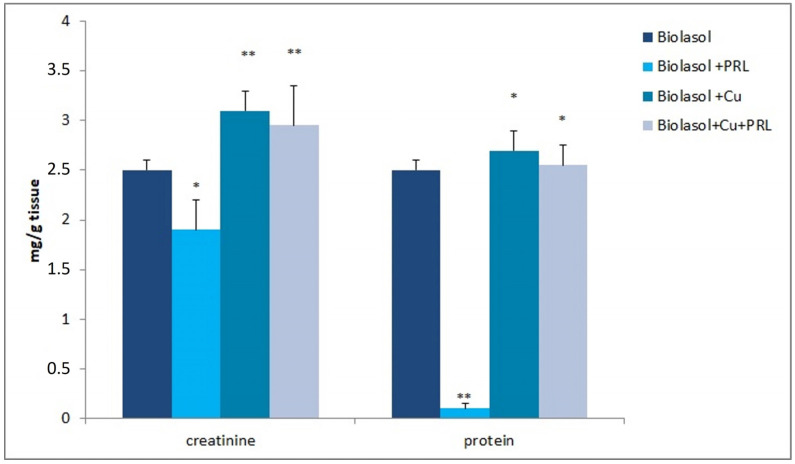
Creatinine and protein concentrations in the kidney homogenates. The values are expressed as mean ± SEM. Data were analysed by one-way ANOVA and Bonferroni post hoc tests; n = 10; * *p* < 0.05; ** *p* < 0.01 compared to the control group (Biolasol).

Figure 7 shows the concentrations of total protein and creatinine in the kidney homogenates. The addition of copper to the Biolasol formulation increased the concentrations of total protein (2.7 ± 0.2 mg/g tissue; control group 2.5 ± 0.1 mg/g tissue, *p* < 0.05) and creatinine (3.1 ± 0.2 mg/g tissue; control group 2.5 ± 0.1 mg/g tissue, *p* < 0.01). The modification of Biolasol fluid with copper and prolactin also increased the analysed indices. Total protein: 2.55 ± 0.2 mg/g tissue, control group 2.5 ± 0.1 mg/g tissue, *p* < 0.05. Creatinine: 2.95 ± 0.4 mg/g tissue, control 2.5 ± 0.1 mg/g tissue, *p* < 0.01).

## 3. Discussion

We tested the effectiveness of copper and the system: Cu^2+^ + PRL, as components of the preservative fluid using Biolasol. This solution is registered and approved for clinical use in Poland. We used a slaughter pig model, the efficacy of which has been established for the testing of different perfusion solutions [26]. Other authors confirm that, for ethical and economic reasons, this is the optimal procedure for qualitative analyses of vital organ function tests in the peri-transplantation period [26,27].

Biolasol is an extracellular fluid with a high concentration of sodium (105 mmol/L) and a low concentration of potassium (10 mmol/L). The substances present in the fluid, which we have described in detail previously [28,29], are supposed to regulate the mechanisms for maintaining normal homeostasis in the graft and restoring normal organ function after transplantation. The antioxidant activity of Biolasol fluid is ensured by the addition of ascorbic acid. Vitamin C scavenges oxygen free radicals, enhances microcirculation, and reduces inflammatory reactions and endothelial permeability [30,31]. Bleilevens et al. [30] suggest that vitamin C may be a beneficial adjunct to clinical renal perfusion procedures. Ascorbic acid is also found in the formulation of Polysol fluid. This is an extracellular fluid developed for hypothermic perfusion [32]. We potentially increased the antioxidant capacity of Biolasol fluid by adding copper ions (1 µg/L) or copper (1 µg/L) and prolactin (0.1 µg/L).

Copper is an antioxidant that exhibits the ability to scavenge and neutralise oxygen free radicals. This bioelement can prevent damage caused by the action of ROS [33,34]. It is a cofactor of superoxide dismutase, a key enzyme of the body’s antioxidant barrier, which catalyses the dismutation reaction of superoxide anion radicals to hydrogen peroxide and molecular oxygen. Consequently, it reduces the effects of oxidative stress by limiting the diffusion of reactive oxygen species released by damaged tissues. Furthermore, it protects against the toxic effects of nitric oxide (NO) by reducing the concentration of pro-inflammatory factors (interleukin-1β/IL-1β and tumour necrosis factor α/TNF-α) [35,36].

The presence of ascorbic acid and Cu^2+^ in the composition of the preservative fluid adversely affected the integrity of renal cell membranes. The increase in AST and LDH activity after 2 h of observation indicates that nephrons were already damaged early in the preservation period (Figure 2 and Figure 3). Although there was a decrease in AST activity in the perfusates after 48 h of preservation (versus 2 h), this was not statistically significant versus the control group, After 48 h 30 min, the increase in AST activity was statistically significantly higher versus Biolasol, which may indicate deeper damage to the cells (including their mitochondrial membranes), as a result of the ongoing inflammatory process. Following ischemia and 48 h of preservation, LDH activity was statistically significantly higher versus the control group, which is due to the increased permeability of cell membranes. The strongest effect of nephron damage was observed in the group of kidneys preserved and flushed with Biolasol + Cu^2+^ + PRL fluid. A marked increase in ALT, AST, and LDH activities was observed after 2 h of preservation (Figure 1, Figure 2 and Figure 3). At other time points, the marker activities were statistically significantly higher versus the control group. The observed effect may be due to the pro-oxidative effect of vitamin C, which reacts with copper to reduce it from Cu^2+^ to Cu^+^ (AscH^−^ + Cu^2+^ → Asc ^•−^ + Cu^+^ + H^+^). The reduced form of Cu^+^ in turn participates in the Haber–Weiss reaction, in which a hydroxyl radical (HO^−^) is generated from hydrogen peroxide. As a consequence, lipid peroxidation and a disruption of protein structure can occur [31,37,38,39]. The above mechanism has been observed in vitro, but there is no clear evidence of such activity in vivo [40]. The damage to the mitochondrial and cytoplasmic membranes of cells observed in our study may also result from the overexposure of the kidney to the copper contained in Biolasol fluid or from the use of too high a copper dose. Cellular copper overload can result in cytotoxicity. Qiao et al. [41] confirmed that during copper ion-induced apoptosis, there is an increase in mitochondrial membrane permeability. Arnal et al. [42] found that copper (depending on concentration and cell type) can trigger cell death by activation of specific protease systems. Guo et al. [43], on the other hand, confirmed that a high dose of copper can cause oxidative stress, apoptosis, DNA damage, and inflammatory responses. Administration of CuSO_4_ to mice at a dose of > 10 mg/kg induced inflammation by increasing the mRNA levels of proinflammatory cytokines such as interleukin-1β (IL-1β), IL-2, IL-12, tumour necrosis factor-α (TNF-α), and interferon-γ (IFN-γ). This resulted in splenic dysfunction. Other authors have suggested [44] that a high supply of copper (16 mg/kg mouse body weight) induces oxidative stress and mitochondrial apoptosis in mouse liver. Increased levels of ROS induced by Cu impair mitochondrial membrane permeability. Histopathological imaging has shown the degeneration and necrosis of hepatocytes. Kelleher et al. [45] confirmed that prolactin stimulates copper transport as a result of increased CTR1 and ATP7A abundance at the plasma membrane. This may result in intracellular Cu^+^ overload [46] and increased damage to cell membrane integrity.

The mean urea values (Figure 4) were high in the Biolasol + Cu^2+^ group, indicating a decrease in renal excretory function. After 2 h of graft storage, the concentration of this parameter was 60 ± 4 mg/dL, while after 48 h, the value was 47 ± 4 mg/dL. This indicates a progression of renal dysfunction. In contrast, in the Biolasol + Cu^2+^ + PRL group, the concentration of this marker was the lowest compared to the other study groups and below the reference interval for pigs (10–30 mg/dL) [47]. It is likely that there was progressive renal tubular damage [48]. Other studies [41,49] have also observed a significant increase in urea concentrations after copper exposure in a rat and pig model. The authors concluded that the bio-element can lead to renal cell death and pathological damage.

A loss of renal function results in a deficit of renal excretory capacity. Water retention, electrolyte disturbances, and increasing acidosis occur [50]. However, analysing Figure 5 and Figure 6, it can be seen that groups B2 and B3 fluids maintain sodium–potassium pump activity at T = 4 °C, influencing the maintenance of sodium and potassium homeostasis.

Figure 7 shows the results of creatinine and protein concentrations in homogenates of isolated pig kidneys. Some authors emphasise the importance of these markers in the assessment of organ failure [51,52]. Creatinine and protein concentrations were elevated in kidney homogenates washed with Biolasol + Cu^2+^ and Biolasol + Cu^2+^ + PRL fluid. Lo et al. [52] suggest that cold storage of kidneys may affect mitochondrial protein homeostasis. An increase in total protein levels correlates with the severity of kidney damage. The increased accumulation of albumin and AGE-albumin (an advanced glycation end product) within the kidney promotes inflammation and oxidative stress leading to renal damage [52]. An increase in creatinine in homogenates in turn increases plasma creatinine levels, indicating a deterioration of renal function [53].

Flushing the kidneys with Biolasol fluid modified with copper or copper and prolactin does not have a protective effect on the kidneys. Only the addition of prolactin to Biolasol significantly improves the biochemical parameters of the grafts in flushing and perfusion models of isolated porcine kidneys. The protective effects of prolactin on the kidneys have been extensively discussed in our previous article [21].

A limitation of our study is that we investigated the potential of copper in renal protection using a preservative fluid containing Cu^2+^ at a concentration of 1 μg/L. Further experimental studies are needed to extend the range of concentrations of this bioelement during analyis.

## 4. Materials and Methods

### 4.1. Ethics Committee Approval

All experimental procedures were carried out under the approval of II Local Ethics Commission for Animal Experiments in Cracow, Poland (No. 1046/2013, approval date: 4 June 2013).

### 4.2. Solution Preservation

Kidneys were preserved through static cold storage (SCS) using Biolasol/modified-Biolasol solution (FZNP, Biochefa, Sosnowiec, Poland). The solution has been registered in Poland as a medical product (Reg.-No. TNP/MDD/0120/3991/2014) and approved for clinical practice. A 1000 mL unit pack of Biolasol fluid contained: dextran 70 kDa (0.7 mmol), glucose (167 mmol), tri-sodium citrate (30 mmol), di-sodium edetate (5 mmol), potassium chloride (10 mmol), fumarate magnesium (5 mmol), sodium bicarbonate (5 mmol), calcium chloride (0.5 mmol), ascorbic acid (0.5 mmol). Biolasol solution has been modified with the addition of Cu^2+^ (1 μg/L) and/or prolactin (0.1 μg/L). Concentrations were established on the basis of previously conducted pilot studies. Copper (II) sulphate pentahydrate was from Sigma Aldrich, Darmstadt, Germany. Pig prolactin was from “FZNP” Biochefa, Sosnowiec, Poland. All substances used in the study were of analytical grade.

### 4.3. Animals and Study Groups

The study used 40 isolated pig kidneys taken from 20 dead Polish Large White pigs (weight 90–110 kg, age 175–180 days). The study used slaughter kidneys, avoiding the use of laboratory animals [26]. The pigs were slaughtered at a local meat-processing plant H.A.M in Radzionków (Poland). Animals were electrocuted with 220 volts before slaughter (in a separate room). European Union legislation was followed (Council Regulation (EC) No. 1099/2009 of 24 September 2009 on the production of animals at the time of killing; directive 86/609/EEC on the use of animals or experimental and other scientific purposes in the EU). After a cardio-circulatory standstill, the kidneys were harvested, separated from the surrounding tissues, and cannulated using a 40 cm Nelaton CH08 catheter (ConvaTec, Deeside, UK). The warm ischemia time was 30 min. Grafts were randomly transferred to polyethylene bags filled with Biolasol (Group A, control) or Biolasol modified fluid (Group B1, B2, B3), respectively, with a volume of 500 mL and a temperature of 4 °C. Kidneys were transported to the Biochefa laboratory (FZNP, Biochefa, Sosnowiec, Poland) in isothermal packaging. The storage time of the kidneys using simple hypothermia was 2 h.

Group A/Control

Kidneys were flushed with Biolasol solution. Cold static storage (4 °C) 2 h and 48 h;

n = 10.

Group B1

Kidneys were flushed with Biolasol solution modified with PRL (0.1 µg/L). Cold

static storage (4 °C) 2 h and 48 h; n = 10.

Group B2

Kidneys were flushed with Biolasol solution modified with Cu^2+^ (1 µg/L). Cold

static storage (4 °C) 2 h and 48 h; n = 10.

Group B3

Kidneys were flushed with Biolasol solution modified with Cu^2+^ (1 µg/L) and PRL

(0.1 µg/L). Cold static storage (4 °C) 2 h and 48 h; n = 10.

### 4.4. Kidney Perfusion and Preservation Protocol

After 2 h of storage, the kidneys were flushed with cold Biolasol/Biolasol-modified fluid at a pressure of 73.5 mm Hg H_2_O, ensuring a continuous flow of the perfusate stream. At two time points (0 and 30 min perfusion 1), perfusate samples were collected from the renal vein for biochemical analysis. After perfusion, the kidneys were stored again for 48 h (the maximum storage time for kidneys in simple hypothermia [54]). After this time, the kidney lavage was repeated (0 and 30 min perfusion 2). Markers for indirect assessment of renal function were determined in the perfusate samples collected [1,2,3]. After a time of 48 h and 30 min, graft samples were taken for biochemical analysis. Protein and creatinine concentrations were analysed in the kidney homogenates.

### 4.5. Biochemical Determinations in Perfusates

The activity of alanine aminotransferase (ALT), aspartate aminotransferase (AST), and lactate dehydrogenase (LDH) was determined using commercial kits from bioMérieux (Lyon, France), as previously reported [1,2,3]. Sodium, potassium, and urea concentrations were determined using commercial kits from Pointe Scientific INC (Marseille, France), as previously reported [1,2,3]. A Marcel S330 spectrophotometer (Marcel, Poland) was used for analyses. Biochemical parameters were determined according to the supplier’s instructions.

### 4.6. Biochemical Analysis in Kidney Homogenates

Samples were homogenised in cold 0.1 M phosphate buffer, pH = 7 (4 °C). Total protein and creatinine concentrations were determined in the supernatants (centrifugation at 15,000 rpm, t = 3 min). Concentrations of biochemical indicators was determined using commercial kits from Pointe Scientific INC (Marseille, France), as previously reported [2].

### 4.7. Statistical Analysis

Results are presented as mean values (n = 10 for each group) with the standard error of the mean (±SEM). The significance of differences between group means was analysed using one-way analysis of variance (ANOVA) and Bonferroni post hoc tests. STATISTICA software version 13.1 (StatSoft, Cracow, Poland) was used for the analyses. Statistical significance was taken as *p* < 0.05.

## 5. Conclusions

We can conclude that the addition of Cu^2+^ at a dose 1 µg/L to the composition of Biolasol fluid affects the generation of ischemic damage in an isolated pig kidney. The intensity of the occurrence of these processes is aggravated by the presence of prolactin at a dose of 0.1 µg/L. We do not recommend the addition of Cu^2+^ or Cu^2+^ + PRL at the concentrations tested in this study to the composition of the preservative solution.

## Data Availability

The data presented in this study are available on request from the corresponding author.

## Sample Availability

Samples of the compounds the Biolasol/modified Biolasol solution are available from the authors.
